# The role of DNA damage repair (DDR) system in response to immune checkpoint inhibitor (ICI) therapy

**DOI:** 10.1186/s13046-022-02469-0

**Published:** 2022-09-07

**Authors:** Congqi Shi, Kaiyu Qin, Anqi Lin, Aimin Jiang, Quan Cheng, Zaoqu Liu, Jian Zhang, Peng Luo

**Affiliations:** 1 Department of Oncology, Zhujiang Hospital, Southern Medical University, 253 Industrial Avenue, Guangzhou, Guangdong 510282 People’s Republic of China; 2 The First School of Clinical Medicine, Southern Medical University, 1838 Guangzhou Avenue North, Guangzhou, Guangdong 510515 People’s Republic of China; 3 The Second School of Clinical Medicine, Southern Medical University, 1838 Guangzhou Avenue North, Guangzhou, Guangdong 510515 People’s Republic of China; 4 Department of Urology, Changhai Hospital, Naval Medical University (Second Military Medical University), Shanghai, People’s Republic of China; 5 Department of Neurosurgery, Xiangya Hospital, Central South University, Changsha, Hunan 410008 People’s Republic of China; 6 National Clinical Research Center for Geriatric Disorders, Xiangya Hospital, Central South University, Changsha, People’s Republic of China; 7 Department of Interventional Radiology, The First Affiliated Hospital of Zhengzhou University, Zhengzhou, Henan People’s Republic of China

**Keywords:** DNA damage repair, DNA damage repair inhibitors, Immune checkpoint inhibitor, Immune checkpoint blockade, cGAS/STING, ATM/ATR/Chk1

## Abstract

As our understanding of the mechanisms of cancer treatment has increased, a growing number of studies demonstrate pathways through which DNA damage repair (DDR) affects the immune system. At the same time, the varied response of patients to immune checkpoint blockade (ICB) therapy has prompted the discovery of various predictive biomarkers and the study of combination therapy. Here, our investigation explores the interactions involved in combination therapy, accompanied by a review that summarizes currently identified and promising predictors of response to immune checkpoint inhibitors (ICIs) that are useful for classifying oncology patients. In addition, this work, which discusses immunogenicity and several components of the tumor immune microenvironment, serves to illustrate the mechanism by which higher response rates and improved efficacy of DDR inhibitors (DDRi) in combination with ICIs are achieved.

## Background

Since its development, immunotherapy has played an important role in the treatment of melanoma [1–3], breast cancer [4], prostate cancer [5], small cell lung cancer [6], and many other types of malignancies [7, 8]. However, the application of an immune checkpoint blockade (ICB) is only provided to a few patients due to its limited efficacy [9–13]. At present, the effect of ICB for cancer patients is primarily predicted by the measurements of programmed cell death 1 ligand 1 (PD-L1) expression levels [14], microsatellite instability (MSI) or mismatch repair-deficient (dMMR) [15], and the tumor mutational burden (TMB) [16–19], to name a few. Apart from the disadvantages associated with the heterogeneity of expression and the uniformities of test platforms [20], evidence has shown that patients may present insensitivity to immunotherapy when PD-L1 expression is high [14, 21, 22]. In addition, although microsatellite instability-high (MSI-H)/dMMR is an acceptable prognostic measure [23, 24], the incidence of dMMR is very low in certain cancers [25–27]. Moreover, the use of TMB values as prognostic markers requires improvement through the continued refinement of subcategories and standard values. Hence, given the shortcomings of current intervention approaches, it is necessary to identify more markers to accurately diagnose and stratify cancer patients of all types.

DNA damage repair (DDR) is a response of cells to DNA damage. Based on the different types of DNA damage, DDR initiates the repair process via different pathways, including mismatch repair (MMR), base excision repair (BER), nucleotide excision repair (NER), homologous recombination repair (HRR), and non-homologous end joining (NHEJ) [28–30]. The high frequency of the DDR gene and pathway alterations exhibited in the samples in the Cancer Genome Atlas (TCGA) PanCanAtlas identifies opportunities to improve cancer therapy [31].

Several studies have recognized a correlation between DDR and ICB [10, 32–34]. The underlying mechanism pertaining to the DDR pathway that affects immune infiltration has also garnered increasing attention. An increase in neoantigen accumulation and induction of the anti-tumor immune response, initiated through the enlargement of somatic mutations and intracellular DNA fragment accumulation, have been recognized to play an active role in DDR dysfunction [35]. In addition to enhancing immunogenicity, the DDR pathways related to tumor cells affects both immune surveillance and the immune response. These can be assessed through both the DNA damage signaling pathway and the accumulation of cytoplasmic DNA subsequently activating the interferon pathway, which is related to the change of T cell infiltration and programmed cell death protein 1 (PD-1)/PD-L1 expression [9, 36]. The negative regulation of DDR inhibition on Treg cells also promotes T cell infiltration [37, 38]. Meanwhile, the role of the DDR pathway in immunotherapy is being evidenced in increasing tumor types [39]. At present, although several reviews have demonstrated the influence of DDR on ICB and immune infiltration [16, 40–44], we illustrate this relationship using specific tumor microenvironment (TME) components. Hence, this work aims to identify, summarize and explore the existing mechanisms and effects of DDR on ICB, so as to facilitate an improved understanding of these specific interactions and the efficacy of related therapies.

## The effect of altered DDR pathway interactions on ICIs

### The predictive effect of altered DDR pathways in ICIs

Change in the tumor DDR pathway is significantly correlated with the response to immune checkpoint inhibitors (ICIs) [24, 45–47] and serves to directly affect patient survival [35, 39, 47]. Among the patients treated with ICB, those with homologous recombination deficiency (HRD) or DDR mutation exhibited better response [13, 47]. For patients with gastrointestinal cancer, advanced urothelial cancer, and metastatic prostate cancer treated with anti-PD-(L)1, DDR changes can lead to the prolongation of overall survival (OS) and progression-free survival (PFS) [47, 48]. Furthermore, as the number of DDR changes increases, the objective response rate (ORR) and the durable clinical benefit (DCB) increased significantly [47, 49]. Therefore, DDR changes can be used as a reliable biomarker in ICI clinical applications [50].

Specific DDR changes can be used to predict different effects. For one, MSI-H/dMMR, which is listed as a predictive biomarker [51, 52], is directly related to greater benefits observed in ICB patients [24, 27, 38, 53]. A well-established close positive relationship between MMR defects and MSI exists [54, 55]. Interestingly, DDR mutations have also been identified in microsatellite stable (MSS) patients [56, 57]. These findings reflect the potential predictive value of DDR mutation as a tool capable of identifying the responder group [56]. In some cases, patients with the homologous recombination (HR) pathway defect have received increased benefits from ICIs [38]. Patients with BAP1 mutant malignant mesothelioma exhibit a significant increase in tumor-infiltrating lymphocytes (TILs) and high expression of immune checkpoint receptors [58]. However, clinical observation of the BRCA1/2 mutation has revealed contradictory results [59, 60]. In an analysis of whole exome sequences recorded in 38 patients diagnosed with melanoma and previously treated with PD-1 inhibitors, the ICB responders expressed augmented states in BRCA2 mutations [61]. However, a large phase 1b study investigating patients with recurrent or refractory ovarian cancer demonstrated that BRCA status was not associated with response to anti-PD-L1 treatment [62]. Cancer presenting with a defective BER pathway may increase the response to ICB. XRCC1 gene expression can be used to divide PD-L1+ and PD-1+ breast cancer patients into groups with different prognoses [63]. POLD1/POLE mutation is applicable to multi-tumor states and is a promising target for achieving accurate stratification [59, 64, 65]. Further evidence can be obtained from a related Phase II clinical trial (NCT03810339) [66]. Lastly, ataxia-telangiectasia mutated (ATM) protein kinase is known to improve the ORR of patients, although it may be related to a shorter OS [22, 49], and combined mutation of TP53 and ATM also constitutes a potential biomarker [67]. The relevant DDR mutations and DDR deficiencies involved in the predication of ICI efficacy are listed in Tables 1 and 2 below, respectively.

**Table 1:** Effectiveness of DDR mutations in predicting ICI efficacy in solid tumors

Clinical endpoint	DDR mutation	ICI	Cancer type	Wild type	Mutation type	Refs
ORR	BRCA1/2	Pembrolizumab	Melanoma	5.0%	12.0%	[58]
	PRKDC	Anti-CTLA-4 antibody+anti-PD-1 antibody	NSCLC	32.3%	66.7%	[68]
	PRKDC	Anti-CTLA-4 antibody	Metastatic melanoma	14.6%	33.3%	[68]
	DDR mutation	Anti-PD-1/PD-L1 antibodies	Urothelial cancer	18.8%	67.9%	[45]
PFS	PRKDC	Anti-CTLA-4 antibody+anti-PD-1 antibody	NSCLC	6.8 m	NR	[68]
OS	POLE/POLD1	ICI	Solid tumors	18.0 m	34.0 m	[66]
	TP53 + ATM	ICI	NSCLC	2.8 m–22.0 m	22.1 m-NR	[67]

*Abbreviations*: *ORR* Objective response rate, *PFS* Progression-free survival, *OS* Overall survival, *NR* Not reached

**Table 2:** Effectiveness of DDR deficiencies in predicting ICI efficacy in solid tumors

Clinical endpoint	DDR deficiency	ICI	Cancer type	Proficiency type	Deficiency type	Refs
ORR	HR	Nivolumab+ipilimumab	Prostate cancer	22.6%	50.0%	[13]
	DDR positive	Nivolumab+ipilimumab	Prostate cancer	23.1%	36.4%	[13]
	DDR positive	ICI	Prostate cancer	20.0%	40.0%	[48]
	MMR	Pembrolizumab	Colorectal cancer	0.0%	40.0%	[24]
PFS	MMR	Pembrolizumab	Colorectal cancer	2.2 m	NR	[24]
OS	MMR	Pembrolizumab	Colorectal cancer	5.0 m	NR	[24]
	HR	Nivolumab+ipilimumab	Prostate cancer	19.0 m	NR	[13]

*Abbreviations*: *ORR* Objective response rate, *PFS* Progression-free survival, *OS* Overall survival, *NR* Not reached

However, the use of individual DDR pathway changes in guiding ICIs treatment remains to be explored [20, 47, 69]. Researchers have established reliable DDR scoring models and effectively analyzed the predictive effect of co-mutation, the DDR gene models of various researchers can effectively predict specific response to ICIs treatment [69–72]. In one case, Yi-Ru Pan et al. selected 18 DDR genes as the defined gene panel and achieved a predicted response rate of about 60% in patients with DDR mutations [73]. Among 102 melanoma patients, 34 patients (33.3%) were classified as the mutated group, and 22 of 34 (64.7%) patients responded to ICIs [73]. The mutant group had significantly longer PFS and OS than the wild-type group [73].

Patients with a co-mutation in multiple DDR pathways demonstrate a good response to ICIs [59, 74]. HRR-MMR, or co-mutation in the HRR-BER pathway, is regarded as a potential biomarker [74–76]. In a study on pembrolizumab, 11 of 34 non-small cell lung cancer (NSCLC) patients were assigned to the co-mutation positive (co-mut+) group [74], in which ORR was 63.6% compared with 21.7% of the co-mutation negative (co-mut-) group. There was also significant PFS improvement in the co-mut + group (median, 4.1 months vs. not reached (NR), *P* = 0.006) [74]. Accordingly, 429 NSCLC patients were treated with atezolizumab, in whom the proportion of DCB in co-mut + patients was twice as high as that in co-mut- patients (56.7% vs. 28.8%), while the ORR was 11.9% higher (26.7% vs. 14.8%) [20]. In addition, even for patients with low PD-L1 expression, co-mut + status improved the overall results [20]. One hundred seventy-four patients presenting with metastatic melanoma and treated with the CTLA-4 antibody exhibited a significantly longer OS in the co-mut + group (median, 32.4 months vs. 10.8 months, *P* = 0.04) [74]. However, no significant difference was observed in the tumor response rate (43.8% vs. 29.5%, *P* = 0.15) [74].

In addition, simultaneous changes in the NOTCH signaling pathway and at least two DDR pathways [77], as well as the combination of TMB and an HR-DDR-positive status [78] may also represent effective biomarkers. Given this, DDR mutations should be considered a useful biomarker for patients receiving ICI treatment.

With regard to the activation level of the DDR pathway, in an analysis of 348 patients with metastatic urothelial carcinoma (UC) who received ICI treatment, the DSSH (DDR ssGSEA enrichment score-high) group was associated with longer OS (hazard ratio = 0.67), whereby a high activation level of the DDR pathway was identified as effective in improving the outcome and prognosis of patients that received ICI treatment [34]. Other studies have also revealed that high expression of the DDR pathway can predict an improved ICI response in patients with gastric cancer (GC) and advanced UC [79, 80]. In addition, the characteristics used to divide patients with hepatocellular carcinoma (HCC) into the DDR activation or inhibition subgroup can effectively help distinguish the clinical and molecular features of HCC, and predict different immunotherapy responses and prognoses [71, 72].

Indeed, even when compared to existing predictive markers such as TMB and PD-L1 expression, DDR mutation demonstrates an excellent prognostic effect [20, 22, 47, 48]. In addition, DDR status predicts different clinical outcomes between immunotherapy and non-immunotherapy [20, 72].

### The combination of DDRi with ICIs in clinical trials

Although a limited number of clinical trials implementing combination therapy have been completed, many researchers have recognized the promising potential of combining DDR inhibitors (DDRi) and ICIs. As a result, a number of trials are currently recruiting, with a majority of them using anti-PD-(L)1 antibodies including durvalumab, pembrolizumab, dostarlimab, and nivolumab, with poly (ADP-ribose) polymerase (PARP) inhibitors (Table 3). In addition, the use of combination therapy seems quite promising for many cancer types. Combination therapy trials for solid tumors have most commonly been conducted on gynecologic tumors, breast cancer, and lung cancer (Table 3), while several clinical trials have also been conducted on gastrointestinal cancers, melanoma, urologic tumors, nasopharyngeal cancer, and osteosarcoma (Table 3).

**Table 3:** Ongoing and completed clinical trials exploring the efficacy of combination DNA targeting and immunotherapy agents

Clinical trial ID	Cancer type	DDR-targeted inhibitor	ICI	Targets	Phase	Clinical endpoint	Status	Refs
NCT04334941	SCLC	Talazoparib	Atezolizumab	PARP+PD-L1	Phase 2	PFS, OS	Recruiting	[81]
NCT04068831	Renal Cell Carcinoma	Talazoparib	Avelumab	PARP+PD-L1	Phase 2	ORR, PFS	Recruiting	[16]
NCT03964532	Advanced Breast Cancer	Talazoparib	Avelumab	PARP+PD-L1	Phase 1 Phase 2	AEs, ORR, PFS, OS	Recruiting	[16]
NCT04678362	Urothelial Carcinoma	Talazoparib	Avelumab	PARP+PD-L1	Phase 2	PFS, OS, DOR	Recruiting	[82]
NCT04173507	Lung Non-Squamous Non-Small Cell Carcinoma	Talazoparib	Avelumab	PARP+PD-L1	Phase 2	ORR, DCR, Toxicity, PFS, OS, DOR,	Active, not recruiting	[16]
NCT03565991	BRCA or ATM Mutant Solid Tumors	Talazoparib	Avelumab	PARP+PD-L1	Phase 2	OR, TTR, PFS, OS, DOR	Active, not recruiting	[83]
NCT02912572	Metastatic Endometrial Cancer	Talazoparib	Avelumab	PARP+PD-L1	Phase 2	PFS, ORR, DOR, irPFS	Recruiting	[16]
NCT04052204	Advanced or Metastatic Solid Tumors	Talazoparib	Avelumab	PARP+PD-L1	Phase 1 Phase 2	DLT, DOR, TTR, PFS, OS	Terminated	[38]
NCT03330405	Solid Tumors	Talazoparib	Avelumab	PARP+PD-L1	Phase 2	DLT, OR, TTR, PFS, OS, DOR	Active, not recruiting	[83]
NCT03637491	Solid tumors	Talazoparib	Avelumab	PARP+PD-L1	Phase1 Phase 2	DLTs, OR, AEs	Terminated Has Results	[83]
NCT04187833	Melanoma	Talazoparib	Nivolumab	PARP+PD-1	Phase 2	BOR, PFS, irOR, irPFS, OS, AEs	Recruiting	[75]
NCT04158336	Solid Tumors	Talazoparib	Pembrolizumab	PARP+PD-1	Phase 1 Phase 2	Safety and Tolerability, MTD, RP2D	Recruiting	[75]
NCT03101280	Advanced Gynecologic Cancers and TNBC	Rucaparib	Atezolizumab	PARP+PD-L1	Phase 1	DLTs, OS, DOR, PFS	Completed	[83]
NCT04276376	Solid Tumors	Rucaparib	Atezolizumab	PARP+PD-L1	Phase 2	ORR	Recruiting	[75]
NCT03824704	Solid Tumors	Rucaparib	Nivolumab	PARP+PD-1	Phase 2	ORR, PFS, DOR	Terminated Has Results	[84]
NCT04624178	Leiomyosarcoma	Rucaparib	Nivolumab	PARP+PD-1	Phase 2	ORR, PFS	Recruiting	[85]
NCT03338790	mCRPC	Rucaparib	Nivolumab	PARP+PD-1	Phase 2	ORR, RR-PSA, PFS	Active, not recruiting Has Results	[83]
NCT03639935	Biliary Tract Cancer	Rucaparib	Nivolumab	PARP+PD-1	Phase 2	PFS, OS	Recruiting	[84]
NCT03572478	Prostate Cancer or Endometrial Cancer	Rucaparib	Nivolumab	PARP+PD-1	Phase 1 Phase 2	DLTs	Terminated Has Results	[83]
NCT02873962	Relapsed Ovarian, Fallopian Tube or Peritoneal Cancer	Rucaparib	Nivolumab	PARP+PD-1	Phase 2	ORR, Safety and Tolerability, PFS, DOR	Recruiting	[84]
NCT03958045	SCLC	Rucaparib	Nivolumab	PARP+PD-1	Phase 2	PFS, DCR, OS, ORR	Recruiting	[16]
NCT03522246	Ovarian Cancer	Rucaparib	Nivolumab	PARP+PD-1	Phase 3	PFS, OS, ORR, DOR, Safety and Tolerability	Active, not recruiting	[83]
NCT02935634	Advanced Gastric Cancer	Rucaparib	Nivolumab Ipilimumab	PARP+PD-1 + CTLA-4	Phase 2	ORR, DOR, AEs	Active, not recruiting	[16]
NCT03869190	Urothelial Cancer	Niraparib	Atezolizumab	PARP+PD-L1	Phase 1 Phase 2	ORR, pCR, PFS, OS	Recruiting	[16]
NCT04313504	Head and Neck Cancer	Niraparib	Dostarlimab	PARP+PD-1	Phase 2	ORR, AEs, PFS, OS	Recruiting	[86]
NCT03955471	Ovarian Cancer	Niraparib	Dostarlimab	PARP+PD-1	Phase 2	ORR, DOR, PFS, OS, DCR	Terminated	[87]
NCT04068753	Cervix Cancer	Niraparib	Dostarlimab	PARP+PD-1	Phase 2	Toxicity, DOR, PFS, OS	Recruiting	[38]
NCT04544995	Neuroblastoma and Osteosarcoma	Niraparib	Dostarlimab	PARP+PD-1	Phase 1	DLTs, PFS, ORR	Recruiting	[88]
NCT04673448	Pancreatic, Ovarian or Fallopian Tube Cancer	Niraparib	Dostarlimab	PARP+PD-1	Phase 1	bOR, PFS, DOR, OS, DC, AEs	Recruiting	[89]
NCT04701307	Lung Small Cell Carcinoma and Neuroendocrine Tumor	Niraparib	Dostarlimab	PARP+PD-1	Phase 2	PFS, ORR, DCR, OS	Recruiting	[90]
NCT04493060	Pancreatic Cancer	Niraparib	Dostarlimab	PARP+PD-1	Phase 2	DCR, ORR, TTNT, OS	Recruiting	[89]
NCT05126342	Ovarian, Peritoneal or Fallopian Tube Cancer	Niraparib	Dostarlimab	PARP+PD-1	Phase 2	ORR, PFS, DCR, OS, TFST	Not yet recruiting	[91]
NCT03016338	Endometrial Cancer	Niraparib	Dostarlimab	PARP+PD-1	Phase 2	CBR, ORR, DOR, PFS	Active, not recruiting	[92]
NCT03307785	Advanced or Metastatic Solid Cancer	Niraparib	Dostarlimab	PARP+PD-1	Phase 1	DLTs, ORR, DOR, DCR	Active, not recruiting Has Results	[83]
NCT04584255	Breast Cancer	Niraparib	Dostarlimab	PARP+PD-1	Phase 2	TILs, pCR	Recruiting	[93]
NCT04940637	Lung Cancer or Mesothelioma	Niraparib	Dostarlimab	PARP+PD-1	Phase 2	PFS, ORR, DCR, DOR, OS	Recruiting	[94]
NCT03651206	Ovarian Cancer and Endometrial Cancer	Niraparib	Dostarlimab	PARP+PD-1	Phase 2 Phase 3	RR, OS, PFS, TTST	Recruiting	[84]
NCT04779151	Solid Tumors	Niraparib	Dostarlimab	PARP+PD-1	Phase 2	ORR	Not yet recruiting	[96]
NCT03602859	Ovarian cancer	Niraparib	Dostarlimab	PARP+PD-1	Phase 3	PFS, OS	Active, not recruiting	[83]
NCT03308942	NSCLC	Niraparib	Dostarlimab Pembrolizumab	PARP+PD-1	Phase 2	ORR, PFS, OS	Completed Has Results	[83]
NCT04508803	Breast Cancer	Niraparib	HX008(Pucotenlimab)	PARP+PD-1	Phase 2	ORR, PFS, OS, CBR, DOR	Recruiting	[86]
NCT03404960	Pancreatic Cancer	Niraparib	Nivolumab Ipilimumab	PARP+PD-1 + CTLA-4	Phase 1 Phase 2	PFS, Safety and Tolerability, DOR, ORR	Active, not recruiting	[51]
NCT04178460	Gastric Cancer, TNBC, Biliary Tract Carcinoma and Endometrial Carcinoma	Niraparib	MGD013(Tebotelimab)	PARP+ PD-1 + LAG-3	Phase 1	Safety and Validity profiles	Terminated	[97]
NCT02657889	TNBC or Ovarian Cancer	Niraparib	Pembrolizumab	PARP+PD-1	Phase 1 Phase 2	DLTs, ORR, Safety and Tolerability, DOR	Completed Has Results	[83]
NCT04475939	NSCLC	Niraparib	Pembrolizumab	PARP+PD-1	Phase 3	PFS, OS, TTP	Recruiting	[98]
NCT04885413	Endometrial Cancer	Niraparib	Sintilimab	PARP+PD-1	Phase 2	ORR, DOR, PFS, DCR	Recruiting	[99]
NCT05162872	Nasopharyngeal Carcinoma	Niraparib	Sintilimab	PARP+PD-1	Phase 2	ORR, DOR, PFS, DCR, OS	Recruiting	[91]
NCT02734004	Advanced Ovarian, Breast, Lung, and Gastric Cancers	Olaparib	Durvalumab	PARP+PD-L1	Phase1 Phase 2	DCR, ORR, Safety and Tolerability	Active, not recruiting	[83]
NCT03334617	NSCLC	Olaparib	Durvalumab	PARP+PD-L1	Phase 2	ORR, DOR, PFS, DCR, OS	Recruiting	[83]
NCT02484404	Recurrent Ovarian, TNBC, Lung, Prostate, and Colon Cancers	Olaparib	Durvalumab	PARP+PD-L1	Phase 1 Phase 2	ORR, RP2D, AEs	Recruiting	[30, 49, 80, 84]
NCT04644289	Epithelial Ovarian Cancer	Olaparib	Durvalumab	PARP+PD-L1	Phase 2	Safety, Feasibility	Recruiting	[100]
NCT03923270	Small-cell Lung Cancer	Olaparib	Durvalumab	PARP+PD-L1	Phase 1	AEs, PFS, OS	Recruiting	[51]
NCT03851614	MMR proficient Colorectal Cancer, Pancreatic Cancer, and Leiomyosarcoma	Olaparib	Durvalumab	PARP+PD-L1	Phase 2	ORR, CBR, PFS	Active, not recruiting	[84]
NCT03534492	Bladder Cancer	Olaparib	Durvalumab	PARP+PD-L1	Phase 2	pCRR, Toxicity	Completed	[101]
NCT03167619	TNBC	Olaparib	Durvalumab	PARP+PD-L1	Phase 2	PFS, OS, ORR	Active, not recruiting	[83]
NCT05222971	Biliary Tract Cancer	Olaparib	Durvalumab	PARP+PD-L1	Phase 2	PFS, OS, Toxicity	Recruiting	[91]
NCT03594396	Advanced or mTNBC	Olaparib	Durvalumab	PARP+PD-L1	Phase 1 Phase 2	pCR, RR	Active, not recruiting	[75]
NCT03544125	TNBC	Olaparib	Durvalumab	PARP+PD-L1	Phase 1	AEs, ORR, DOR, PFS, OS	Completed	[16]
NCT03801369	mTNBC	Olaparib	Durvalumab	PARP+PD-L1	Phase 2	ORR, OS	Recruiting	[16]
NCT03737643	Ovarian cancer	Olaparib	Durvalumab	PARP+PD-L1	Phase 3	PFS, pCR, ORR	Recruiting	[16]
NCT03459846	Bladder Cancer	Olaparib	Durvalumab	PARP+PD-L1	Phase 2	PFS, OS, DOR, ORR	Active, not recruiting	[83]
NCT03991832	Solid Tumors	Olaparib	Durvalumab	PARP+PD-L1	Phase 2	ORR, PFS, OS	Recruiting	[102]
NCT02882308	Head and Neck Squamous Cell Carcinoma	Olaparib	Durvalumab	PARP+PD-L1	Phase 2	ORR, pCR	Completed	[84]
NCT03772561	Advanced Solid Tumors	Olaparib	Durvalumab	PARP+PD-L1	Phase 1	ORR	Recruiting	[84]
NCT03775486	Lung Cancer	Olaparib	Durvalumab	PARP+PD-L1	Phase 2	PFS, OS, DOR, ORR	Active, not recruiting	[16]
NCT05209529	TNBC	Olaparib	Durvalumab	PARP+PD-L1	Phase 2	OS, pCR	Not yet recruiting	[91]
NCT04538378	Lung Cancer	Olaparib	Durvalumab	PARP+PD-L1	Phase 2	ORR, PFS, Safety and Tolerability, OS	Recruiting	[103]
NCT04336943	Prostate Cancer	Olaparib	Durvalumab	PARP+PD-L1	Phase 2	Undetectable PSA, AEs	Recruiting	[104]
NCT03951415	Endometrial Cancer	Olaparib	Durvalumab	PARP+PD-L1	Phase 2	PFS, ORR, OS	Active, not recruiting	[44]
NCT04169841	Solid Tumors	Olaparib	Durvalumab, Tremelimumab	PARP+PD-L1 + CTLA-4	Phase 2	PFS	Recruiting	[16]
NCT02953457	Recurrent or Refractory Ovarian, Fallopian Tube or Primary Peritoneal Cancer with BRCA Mutation	Olaparib	Durvalumab Tremelimumab	PARP+PD-L1 + CTLA-4	Phase 2	DLTs, PFS, OS	Active, not recruiting	[84]
NCT03699449	Recurrent Ovarian Cancer	Olaparib	Durvalumab	PARP+PD-L1	Phase 2	ORR, OS, PFS	Recruiting	[16]
NCT04306367	Bile Duct Cancer	Olaparib	Pembrolizumab	PARP+PD-1	Phase 2	RR, DOR, PFS, OS, Safety and Tolerability	Recruiting	[105]
NCT03810105	Prostate Cancer	Olaparib	Durvalumab	PARP+PD-L1	Phase 2	PSA detection	Recruiting	[16]
NCT02546661	Bladder Cancer	Olaparib	Durvalumab	PARP+PD-L1	Phase 1	Safety and Tolerability, ORR, DCR, PFS, DOR, OS	Active, not recruiting	[83]
NCT04269200	Endometrial Cancer	Olaparib	Durvalumab	PARP+PD-L1	Phase 3	PFS, OS, ORR, DOR, TFST	Recruiting	[16]
NCT03741426	Renal Cancer	Olaparib	Durvalumab	PARP+PD-L1	Phase 2	Proof-of-Mechanism, AEs	Recruiting	[16]
NCT04641728	Cervical Cancer	Olaparib	Pembrolizumab	PARP+PD-1	Phase 2	ORR, DOR, DRR, PFS	Active, not recruiting	[106]
NCT04380636	NSCLC	Olaparib	Pembrolizumab	PARP+PD-1	Phase 3	PFS, OS, ORR, DOR, AEs	Recruiting	[44]
NCT04483544	Cervical Cancer	Olaparib	Pembrolizumab	PARP+PD-1	Phase 2	irORR, PFS, TEAEs, DOR	Recruiting	[44]
NCT02861573	mCRPC	Olaparib	Pembrolizumab	PARP+PD-1	Phase 1 Phase2	AEs, ORR, DCR, OS, DOR, PFS	Recruiting	[83]
NCT04191135	Advanced TNBC	Olaparib	Pembrolizumab	PARP+PD-1	Phase 2	PFS, OS	Active, not recruiting	[93]
NCT04123366	Solid Tumors	Olaparib	Pembrolizumab	PARP+PD-1	Phase 2	ORR, DOR, PFS, OS, AEs	Recruiting	[16]
NCT04548752	Pancreatic Cancer	Olaparib	Pembrolizumab	PARP+PD-1	Phase 2	PFS, OS, ORR, AEs	Recruiting	[89]
NCT03834519	Prostate Cancer	Olaparib	Pembrolizumab	PARP+PD-1	Phase 3	OS, rPFS	Active, not recruiting	[107]
NCT04666740	Metastatic Pancreatic Ductal Adenocarcinoma	Olaparib	Pembrolizumab	PARP+PD-1	Phase 2	PFS, OS	Recruiting	[108]
NCT05033756	Breast Cancer	Olaparib	Pembrolizumab	PARP+PD-1	Phase 2	ORR, DOR, PFS, OS, Safety and Toxicity	Not yet recruiting	[91]
NCT03976362	Squamous NSCLC	Olaparib	Pembrolizumab	PARP+PD-1	Phase 3	PFS, OS, AEs	Active, not recruiting	[51]
NCT04633902	Metastatic Melanoma	Olaparib	Pembrolizumab	PARP+PD-1	Phase 2	ORR, PFS, OS, Safety and Toxicity	Recruiting	[90]
NCT05201612	Metastatic Colorectal Cancer	Olaparib	Pembrolizumab	PARP+PD-1	Phase 2	ORR, PFS, OS, DCR, DOR	Not yet recruiting	[91]
NCT05093231	Pancreatic Cancer	Olaparib	Pembrolizumab	PARP+PD-1	Phase 2	ORR, Safety and Toxicity, PFS, DOR, OS	Not yet recruiting	[109]
NCT03740165	Ovarian Cancer	Olaparib	Pembrolizumab	PARP+PD-1	Phase 3	PFS, OS	Active, not recruiting	[16]
NCT05203445	Breast Cancer	Olaparib	Pembrolizumab	PARP+PD-1	Phase 2	MRGB	Recruiting	[91]
NCT05156268	Endometrial Carcinosarcoma	Olaparib	Pembrolizumab	PARP+PD-1	Phase 2	ORR	Recruiting	[91]
NCT03025035	Breast Cancer	Olaparib	Pembrolizumab	PARP+PD-1	Phase 2	ORR, PFS, OS, CBR, DOR	Recruiting	[93]
NCT04417192	Ovarian Cancer	Olaparib	Pembrolizumab	PARP+PD-1	Phase 2	ORR, CRS, OS, PFS	Recruiting	[100]
NCT02485990	Epithelial Ovarian, Fallopian Tube or Primary Peritoneal Cancer	Olaparib	Tremelimumab	PARP+CTLA-4	Phase 1	AEs, MTD, PFS, OS, ORR, DCR, DOR	Terminated	[16]
NCT04034927	Recurrent Ovarian, Fallopian Tube or Peritoneal Cancer	Olaparib	Tremelimumab	PARP+CTLA-4	Phase 2	PFS, DLT, OS, AEs	Active, not recruiting	[16]
NCT02571725	BRCA mutant Ovarian Cancer	Olaparib	Tremelimumab	PARP+CTLA-4	Phase 1 Phase 2	RP2D, ORR, PFS	Active, not recruiting	[75]
NCT02660034	Solid Tumors	Pamiparib	Tislelizumab	PARP+PD-1	Phase 1	AEs, DLT, ORR, DOR, PFS, OS, DCR, CBR	Completed Has Results	[83]
NCT03061188	Solid tumors	Veliparib	Nivolumab	PARP+PD-1	Phase 1	MTD, AEs, ORR, CBR, PFS	Active, not recruiting Has Results	[75]
NCT04216316	Advanced Squamous Cell NSCLC	Berzosertib	Pembrolizumab	ATR + PD-1	Phase 1 Phase 2	RP2D, PFS, OS	Recruiting	[110]
NCT04266912	Solid Tumors	Berzosertib	Avelumab	ATR + PD-L1	Phase 1 Phase 2	AEs, DLTs, MTD, CBR, RR, PFS, OS	Recruiting	[75]
NCT05061134	Melanoma	Ceralasertib	Durvalumab	ATR + PD-L1	Phase 2	ORR, DOR, TTR, PFS	Recruiting	[111]
NCT03334617	NSCLC	Ceralasertib	Durvalumab	ATR + PD-L1	Phase 2	ORR, DCR, DOR, PFS, OS	Recruiting	[75]
NCT04298008	Biliary Tract Cancer	Ceralasertib	Durvalumab	ATR + PD-L1	Phase 2	DCR, ORR, PFS, OS, DOR	Recruiting	[112]
NCT03780608	Gastric Adenocarcinoma and Malignant Melanoma	Ceralasertib	Durvalumab	ATR + PD-L1	Phase 2	ORR	Active, not recruiting	[16]
NCT03833440	NSCLC	Ceralasertib	Durvalumab	ATR + PD-L1	Phase 2	DCR, ORR, PFS, OS, DOR	Recruiting	[16]
NCT02664935	NSCLC	Ceralasertib	Durvalumab	ATR + PD-L1	Phase 2	OR, PFS, DCB	Active, not recruiting	[44]
NCT02264678	Solid Tumors	Ceralasertib	Durvalumab	ATR + PD-L1	Phase 1 Phase 2	Safety and Tolerability, Cmax, Tmax	Recruiting	[75]
NCT04095273	Solid Tumors	Elimusertib	Pembrolizumab	ATR + PD-1	Phase 1	TEAEs, DLTs, RP2D	Recruiting	[110]
NCT03495323	Advanced Solid Tumors	Prexasertib	LY3300054	CHK1 + PD-L1	Phase 1	Toxicity	Completed	[113]
NCT02546661	Bladder Cancer	Adavosertib	Durvalumab	WEE1 + PD-L1	Phase 1	AEs, ORR, DCR, PFS, OS, DOR	Active, not recruiting	[114]
NCT02617277	Solid Tumors	Adavosertib	Durvalumab	WEE1 + PD-L1	Phase 1	DLTs, TEAEs, ORR, PFS	Active, not recruiting	[51]

*AE* Adverse event, *bOR* Best objective response, *BOR* Best overall response, *CBR* Clinical benefit rate (CBR = CR + PR + SD), *Cmax* Maximum observed plasma concentration, *CRS* Chemotherapy response score, *DC* Disease control, *DCB* Durable clinical benefit, *DCR* Disease control rate, *DLT* Dose limiting toxicity, *DOR* Duration of response, *DRR* Durable response rate, *irOR* Immune-related overall response, *irORR* Immune-related overall response rate, *irPFS* Immune-related progression free survival, *mCRPC* Metastatic castration-resistant prostate cancer, *MMR* Mismatch repair, *MRGB* MRI-guided biopsy, *MTD* Maximum tolerated dose, *mTNBC* Metastatic triple-negative breast cancer, *NSCLC* Non-small cell lung cancer, *OR* Objective response, *ORR* Overall response rate, *OS* Overall survival, *pCR* Pathologic complete response, *pCRR* Pathological complete response rate, *PFS* Progression-free survival, *PSA* Prostate-specific antigen, *rPFS* Radiographic progression-free survival, *RP2D* Recommended phase II dose, *RR* Response rate, *RR-PSA* Prostate-specific antigen response rate, *SCLC* Small cell lung cancer, *TEAEs* Treatment emergent adverse events, *TFST* Time to initiation of the first subsequent anticancer therapy, *TILs* Tumor-infiltrating lymphocytes, *Tmax* Time to observed Cmax, *TNBC* Triple-negative breast cancer, *TTNT* Time to next treatment, *TTP* Time to progression, *TTR* Time to tumor response, *TTST* Time to subsequent treatment

PARP inhibitors (PARPi) are primarily used in the treatment of HRD tumors [38, 115, 116]. The synthetic lethality strategy entails a simultaneous inactivation of two functional genes leading to cell death. It is generally understood that PARPi functions by blocking the BER pathway, or other repair factors, through its inhibition of the core DNA damage sensors and signal transducers, PARP1/2 enzymes [84, 116]. As a result, cells are unable to repair single-stranded DNA damage. After the transformation of single-strand damage into double-strand damage, unlike normal cells, HR-deficient cells cannot be repaired through the HR pathway [117, 118]. Moreover, PARP activity inhibition can cause the NHEJ repair process to be more error prone [119]. Now, PARPi can be used in patients with BRCAness beyond germline BRCA1/2 mutations [116, 120]. In addition, some PARPi have been found to trap PARP1 on DNA-PARP complexes, which stalls the DNA replication fork and interferes with the DNA replication process [84]. Indeed, the trapping potency of many PARPi is roughly comparable to their related cytotoxic effects [118, 120].

PARPi can enhance anti-tumor immunity induced by anti-PD-L1 therapy, and its non-model specificity has been scientifically proven [9, 121–123]. The overall tolerance of 35 patients diagnosed with metastatic castration-resistant prostate cancer after combined treatment with olaparib and durvalumab was good, with a reported overall response rate of 14% and a disease control rate (PR + SD) of 71% [124]. A similar type of PARPi named niraparib combined with pembrolizumab, was observed in the treatment of triple-negative breast cancer (TNBC) and ovarian cancer, which also proved efficient with regard to anti-tumor activity in Phase I and Phase II trials [125]. Moreover, the ORR of patients with a BRCA1/2 mutation is significantly higher [125]. Further, the 1a/b Phase trial of oral PARPis, pamiparib and tislelizumab, demonstrated good tolerance [126]. Therefore, when compared individually with PARPi or a PD-(L)1 monoclonal antibody, the combination of the two can enhance tumor control. In another study, Konstantinopoulos et al. showed that, for patients who lack a BRCA mutation and have platinum-resistant ovarian carcinoma, the combination of an anti-PD-1 antibody and niraparib (ORR, 19%) appears to improve efficacy when compared with a single-agent PARP inhibitor (ORR, about 5%) or an anti-PD-1 antibody (ORR, 4–10%) [125].

Also, targeting cytotoxic T-lymphocyte-associated protein 4 (CTLA-4), PARPi combined with anti-CTLA-4 antibodies has demonstrated similar effects [127, 128]. Apart from immune checkpoint blockade therapy, chimeric antigen receptor T cell (CAR-T) immunotherapy has not been applied to solid tumors. However, olaparib has been shown to enhance CAR-T in the treatment of renal cancer and breast cancer models [129, 130].

At present, several other DDR inhibitors are also being actively implemented in clinical trials (Table 3). It has been shown that the combination of checkpoint kinase (CHK) inhibition and ICB is effective in the treatment of various cancers [9, 113, 131]. The WEE1 inhibitor, when combined with both radiation and a PD-1 blockade, can enhance the cytotoxic activity mediated by CD8 + T cells [132]. In addition, to further aggravate DNA damage and other mechanisms affiliated with the killing of tumor cells, some clinical trials have investigated triple therapy of two DDR inhibitors combined with ICIs, which have also yielded positive results [112, 133].

## The mechanism of altered DDR pathway affecting the efficacy of ICIs

**Fig. 1:**
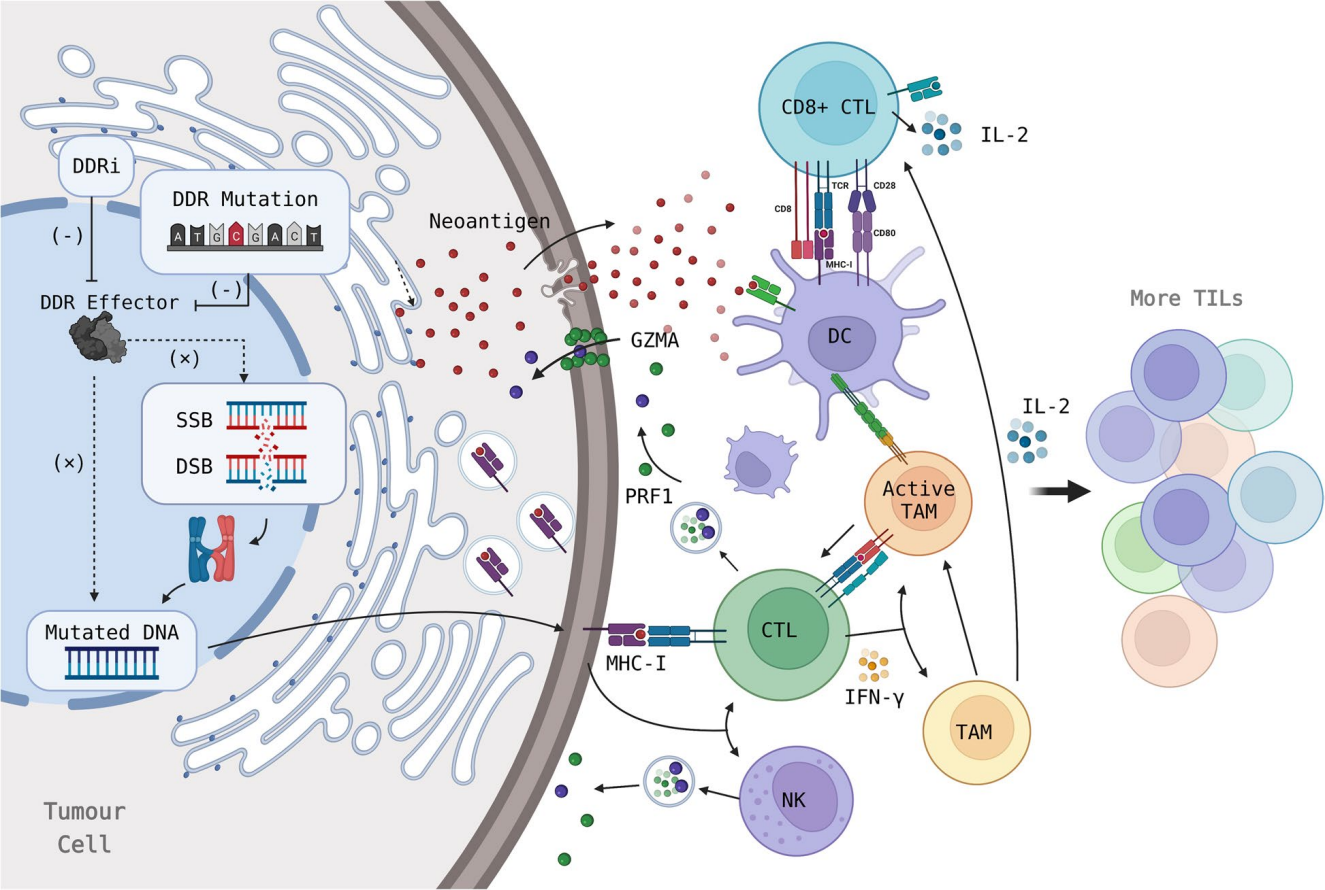
DDR pathway disorders contribute to immune recognition and tumor killing by increasing tumor immunogenicity. DDR mutation/inhibition impedes damaged DNA repair, enlarges chromosomal variation, and thus increases the levels of the tumor mutational burden (TMB) and neoantigen load (NAL). This subsequently activates CTLs and NK cells to exert anti-tumor activity via upregulating MHC-I and the antigen presentation process of APCs. This includes DCs and TAMs. At the same time, more tumor infiltrating lymphocytes (TILs) are recruited. (APC: Antigen-presenting cell; CTL: Cytotoxic T lymphocyte; DC: Dendritic cell; DDR: DNA damage repair; DSB: DNA double strand break; GZMA: Granzyme A; IL-2: Interleukin-2; NK cells: Natural killer cells; PRF1: Perforin 1; SSB: DNA single strand break; TAM: Tumor-associated macrophage)

**Fig. 2:**
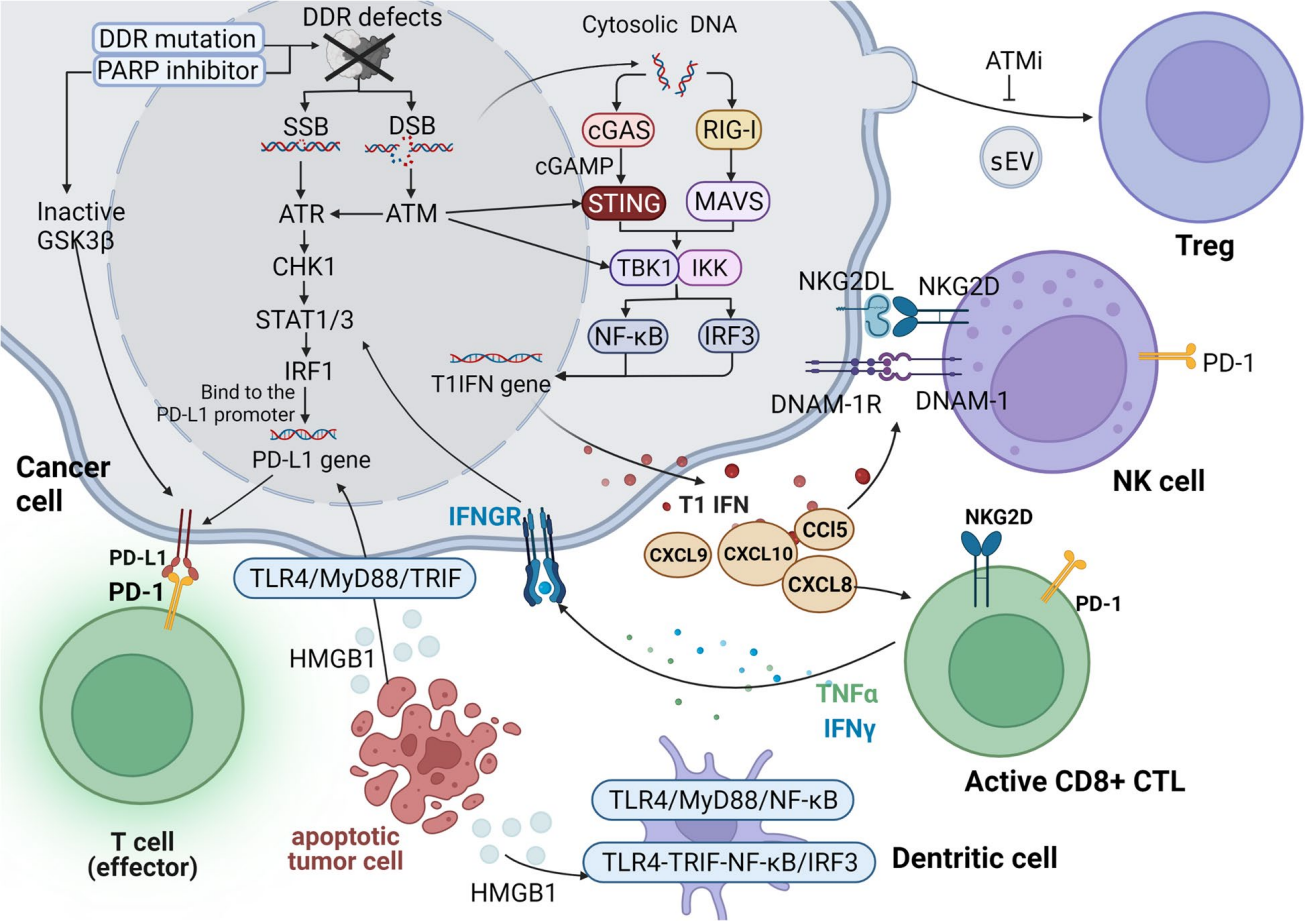
The mechanism by which DDR affects PD-L1 expression and TME in tumors. In tumors, the defectiveness or inhibition of the DDR can lead to the accumulation of DNA damage, whereby double-stranded DNA and single-stranded DNA both accumulate in cytoplasm. Cytoplasmic DNA activates the cGAS/STING and RIG-I/MAVS pathways and eventually the type I interferon (IFN) pathway, ultimately recruiting both chemokines and immune cells (such as T cells, NK cells, and DCs). Specifically, STING promotes the phosphorylation and nuclear translocation of type I IFN transcriptional regulatory factors TBK1 and IFN regulator 3 (IRF3), while also activating the NF-κB pathway that interacts with IRF3. RIG-I can be considered an important participant in the immune activation of cancers presenting with genomic instability, as it can be activated via DNA, and combined with the adapter molecule MAV, which then activates IKK and/or TBK1 when stimulated, and finally activates the type I IFN pathway through downstream transcription factors. The activated TIL releases IFNγ, which acts on tumor cells and mediates STAT1/3-dependent PD-L1 upregulation. The ATM/ATR/Chk1 pathway can also induce PD-L1 expression. ATM can directly activate and participate in STING-mediated downstream pathways, and PARPi can promote PD-L1 expression by downregulating GSK3β. The release of the HMGB1 protein from dying tumor cells can bind TLR-4 on the surface of both DCs and macrophages in order to induce INF-β (TRIF) signal transduction, which subsequently activates IRF3 and NF-κB pathways. In addition, TLR4 recruits MyD88 and activates the NF-κB pathway to promote the transcription and secretion of various pro-inflammatory factors. Following this sequence of events, these factors serve to promote DC activation and trigger an immune response. HMGB1 can also upregulate PD-L1 expression in adjacent surviving tumor cells via TLR4/MyD88/TRIF signaling. In addition, ATM inhibitors can inhibit the induction of Tregs by tumor cell-derived small extracellular vesicles (sEV). (ATM: Ataxia telangiectasia mutated protein; ATR: Ataxia telangiectasia and Rad3-related protein; CCL5: C-C motif chemokine ligand 5; cGAMP: Cyclic GMP-AMP; cGAS: Cyclic GMP-AMP synthase; CHK1: Checkpoint kinase 1; CTL: Cytotoxic CD8+ T cell; CXCL10: C-X-C motif chemokine ligand 10; DDR: DNA damage response; DNAM-1: DNAX accessory molecule 1; DSB: Double-strand break; GSK-3β: Glycogen synthase kinase-3β; HMGB1: High mobility group box 1; IFNγ: Interferon-γ; IFNGR: Interferon gamma receptor; IKK: IκB kinase; IRF1: Interferon regulatory factor 1; IRF3: Interferon regulatory factor 3; MAVS: Mitochondrial antiviral signaling protein; MyD88: Myeloid differentiation factor 88; NF-κB: Nuclear factor kappa-B; NKG2D: Natural-killer group 2, member D; NKG2DL: NKG2D ligand; PARP: Poly-ADP-ribose polymerase; PD-1: Programmed cell death protein 1; PD-L1: Programmed death-ligand 1; RIG-I: Retinoic acid-inducible gene I; sEV: Small extracellular vesicles; SSB: Single-strand break; STAT1/3: Signal transducer and activator of transcription 1/3; STING: Stimulator of interferon genes; T1IFN: Type I interferon; TBK1: TANK binding kinase-1; TLR4: Toll-like receptor 4; TNFα: Tumor-necrosis factorα; Tregs: Regulatory T cells; TRIF: TIR-domain-containing adaptor inducing interferon-β)

The success of immunotherapy is based on the characteristics of tumor cells and the ability to initiate an anti-tumor immune response and, likewise, the efficacy of ICIs is directly related to the functions of the immune microenvironment [134, 135]. The altered DDR pathway in tumors, which results from DDR gene defects or DDRi, directly affects the efficacy of ICIs by affecting immunogenicity (Fig. 1), immune cell infiltration, and the related regulating molecules (Fig. 2) [34, 38, 51, 136].

DDR pathway dysfunction in tumors is capable of activating an immune response, reshaping the immune environment, and is ultimately beneficial to the effectiveness of ICIs. The primary mechanisms involved can be described as follows: (1) increasing the level of neoantigen and tumor immunogenicity, which is beneficial to the immune recognition process in antigen presentation; (2) the activated cyclic GMP-AMP synthase (cGAS) stimulator of interferon genes (STING) pathway triggers innate immunity and enhances the recruitment and tumor infiltration of T cells; (3) ATM/ataxia telangiectasia and Rad3-related protein (ATR)/Chk1 signals cascade to regulate the tumor microenvironment (TME), such as up-regulating PD-L1; and (4) other ways to enhance immunogenic cell death that help activate adaptive immunity [51, 76, 136, 137].

### Alteration of the DDR pathway affects immunogenicity

The fidelity of DNA repair affects the generation of genome mutation [76]. DDR defects in tumor cells, or DDR inhibitors, can aggravate DNA damage and genomic instability (Fig. 1), leading to an increase in MSI, TMB, and neoantigen load (NAL) [38, 138–140]. Previous research has described the ability of DDR alterations in predicting high TMB and high MSI [64, 73, 76, 141–144]. In a NSCLC and melanoma cohort, a high TMB was the result of a DDR mutation accumulation [74]. Compared to HR wild-type tumors, the average TMB of HR-mutated tumors is higher in subtypes, MSI-H/dMMR is most common, and the result of a higher TMB is independent of microsatellite status [145]. Moreover, tumor samples presenting with different altered DDR pathways are also observed alongside an increase of NAL [47]. Notably, dMMR tumors have displayed the ability to enahnce the expression of mutation-related neoantigens [146]. In the other study, tumor samples with any mutation in NHEJ, HR, or DNA damage signaling genes have shown high NAL expression [76, 147]. Furthermore, combined mutation of multiple pathways indicates a higher level of NAL, which may be related to impaired availability of alternative repair pathways [76]. Tumors with POLE/POLD mutation have shown higher TMB and NAL, alongside improved responses to ICIs [59, 148, 149], while the DDR pathway characteristics established by Lou et al. are positively correlated with both the TMB and NAL [80]. Meanwhile, in addition to dMMR tumors, tumors with low BER/single-strand break repair (SSBR) gene expression have shown high MSI and neoantigen production [76, 150].

A high TMB can predict MSI-H and vice versa [64, 69, 151]. It should be noted that although MSI-H/dMMR is associated with TMB-H, DDR genes are still associated with a high TMB after MSI-H/dMMR is excluded [69]. As for the relationship between NAL and TMB, previous studies have proven that high TMB can cause an increase in neoantigens [137].

The elevation of MSI, TMB, and NAL are all beneficial to therapeutic immune recognition, and thus affect the response of the tumor to ICIs (Fig. 1). DDR inhibitors, such as PARPi, may increase both the mutation load and the number of neoepitopes, thereby increasing the response rate to ICIs [152]. The underlying mechanism of action is that a high TMB can increase the expression of immunogenic peptides [69, 73, 118, 153–156]. Specifically, an increased mutation load enhances CD8 + T cell infiltration (Fig. 1) [69, 101, 144], and the intracellular accumulation of DNA fragments furthers induces innate and adaptive immune activation [78]. An increase of NAL can also lead to active immune stimulation of the TME. For example, NAL can actively stimulate TILs to release IFN-γ (Fig. 1) [52]. dMMR tumors are highly sensitive to ICIs, and the objective response rate in CRC is recorded to be between 30 and 50% [24, 157, 158]. In examining tumor tissue, the effective rate of ICB was found to be higher in the TMB-H and MSI-H state than in the TMB-low or MSI-low/MSS state [56, 154].

However, certain cancer cells can also escape immune surveillance [84, 159]. This evasive ability may be attributed to the fact that mutation accumulation caused by DDR defects can also increase the level of PD-L 1[160, 161]. A dMMR/MSI-H tumor or HR-mutated tumor exhibits a strong expression of immune checkpoint ligand [15, 145, 146]. Much like the mechanism behind neoantigen production, the PD-L1 upregulation in tumor cells is related to both the TMB and the MSI. In addition, DDR deficiency upregulates the release of interferons (IFN) by neoantigen-activated T cells, which also promotes the expression of PD-L1 in the tumor environment [162].

DDR mutation can also affect the response to ICIs in ways that are independent of the TMB and MSI [47, 56]. For instance, the increase of the TMB is only one of the ways in which DDR defects lead to genomic instability. The genome of small cell lung cancer (SCLC), specifically characterized by the joint deletion of RB1 and TP53, is known to potentially cause inherent genomic instability, which is independent of the mutation burden [121]. In breast cancer, BRCA1 inactivation is accompanied by an increase of T cells [4, 147, 152, 163]. However, defects observed in TILs were detected in BRCA2-mutated breast cancers [163, 164]. Furthermore, in addition to the above regarded antigenicity, the adjuvanticity of immunogenic cell death (ICD) induction remains an important feature [165]. In this regard, before tumor-specific antigens elicit adaptive immunity, specific microorganism-associated molecular patterns (MAMPs) already activate pattern recognition receptors (PRRs) and initiate the cancer killing effect of the innate immune system. Such signals can be delivered to PRRs via the damage-associated molecular patterns (DAMPs) of dead cells [165, 166].

### Alteration of the DDR pathway and immune infiltration

The poor response of some patients to ICIs is directly related to insufficient production and activity of anti-tumor CD8 + T cells. The aggravation of tumor hypoxia is recognized as another major obstacle in improving the efficacy of ICIs, which is related to the regulation of the TME via immunosuppressive microenvironment-related macrophages, regulatory T cells (Tregs), and so on [75, 93, 167–169]. Therefore, the effect of DDR on immune cell infiltration is likely to facilitate effective ICI treatments.

The effect of the DDR pathway activation level on immune infiltration is complex. The increased immune cell infiltration of gastric cancer is unexpectedly correlated with a low DDR characteristic score. However, insight into the relative abundances of immune cell subpopulations suggests that TILs are positively correlated with DDR pathway signature scores. Therefore, patients with a high score demonstrate a higher proportion of TIL, which corresponds to a better prognosis [80]. In HCC, the proportion of activated immune cells increases significantly in patients with a high expression of DDR-related genes (DDR2) [71, 72]. The subtype of activated DDR has demonstrated significantly increased expression of the immune checkpoint, and its immune score was significantly higher than that of the low expression group [72]. In addition, it is important to note that mutations or defects in the DDR signaling pathway genes are associated with altered immune cell infiltration.

#### T cell

DDR inhibitors, such as PARPi and CHK1 inhibitor (CHK1i) enhance T-cell infiltration [113]. Olaparib alone has been shown to increase the number of intratumoral effector CD4+ and CD8+ T cells, it can also decrease the expression of PD-1/Tim-3 and PD-1/Lag-3 co-inhibitory receptors on CD8+ T cells [170]. ICIs alone do not significantly alter the abundance and function of effector/memory T cells within BRCA1-deficient models [128]. When PARPi was used in combination with CTLA-4 mabs (but not with PD-1 mabs), CD4+ and CD8+ T cells, in addition to IFN-γ levels, were all significantly elevated [128]. However, another study demonstrated no significant change in CD8+ CTL infiltration with olaparib alone, while the combination of olaparib and PD-L1 blockade increased CTL infiltration [9].

Moreover, mutations in the DDR signaling pathway are associated with an increase of T cell infiltration in the TME [56], although the infiltration components are heterogeneous in different tumors [22, 61, 63, 143, 147, 152, 153, 171–174]. To name a few, gastrointestinal cancer patients with more than 2 DDR mutations display a greater infiltration of CD4+/CD8+ T cells [47], while DDR gene somatic mutations in ovarian cancer patients are related to higher levels of Th1 cell infiltration [143].

Alterations of the DDR pathway in tumors promote effector T cell and other cell infiltration in the TME through activating the IFN pathway. DNA damage exacerbated by altered DDR pathways can activate cGAS/STING, ATR/SRC/TBK1, ATM/ATR/Chk1, and other pathways, all of which then activate the downstream IFN pathway (Fig. 2) [33, 171]. In tumor cells, STAT1 and/or STAT3 can initiate typical IFN1 signals. This promotes systemic immune responses and regulates various anti-tumor immune components including T cells, NK cells, and DC, as well as effector molecules (Fig. 2) [38, 44, 84, 171]. The downstream target TIL then collects chemokine genes CXCL9, CXCL10, CXCL11, and CD8 + T cell activation markers (Fig. 2) [171], all of which contribute to a successful anti-tumor immune response. Thus, aside from its beneficial effect on adaptive immunity, IFN1 demonstrates an important functional role in driving the adjuvanticity of immunogenic cell death [165]. PARPi treatment has also been shown to increase the level of chemokines through the cGAS/STING pathway, induce the activation and function of both CD4 + T cells and CD8 + T cells, and increase the level of tumor necrosis factor α (TNF-α) in the TME (Fig. 2) [38, 124, 170, 175].

However, IFN-γ produced by activated TIL often acts as a double-edged sword in anti-tumor immune response. On the one hand, IFN-γ displays the positive anti-tumor immune effect of up-regulating the MHC, which in turn contributes to antigen presentation [52]. On the other hand, IFN-γ exposure increases PD-L1 expression through the JAK-STAT pathway, thus inducing negative feedback and adaptive resistance [52, 176]. IRF1 combined with the PD-L1 promoter enhances PD-L1 transcription through IFN-γ induction (Fig. 2) [162].

##### CD8+ T cell

DDR inhibition enhances CTL function. For example, in one study, PARP/CHK1 inhibition in SCLC patients increased CTL infiltration [131]. While augmented production of IFN-γ and TNF-α by CD8+ T cells was observed in patients after olaparib treatment [170]. Moreover, the active inhibition of ATM prevented Treg-induced CD8+ T cell senescence [177].

A non-neoantigen-dependent increase in CTL infiltration may be associated with the activation of the ATM gene [178]. Phosphorylated ATM was identified as a driving factor of cytokine production that can increase tumor cell-derived levels of CCL5, CXCL9, CXCL10, and IL16, leading to increased CD8+ CTL infiltration [178]. Moreover, in various cancer types, positive CTL score correlation with ATM protein, and neoantigens levels have been identified as being largely mutually exclusive [178]. Ciriello et al. appropriately classified cancers into either class C clusters or class M clusters [179]. Class M tumor profiles exhibit neoantigen-dependent CTL, whereas class C tumor profiles often indicate dependence on ATM [178].

##### CD4+ T cell

DDR inhibitors promote Th1 infiltration. In tumors, continuous low-level DNA stimulation incites the infiltration of inhibitory immune cells [38]. This transformation promotes chronic inflammation, immunosuppression, and tumor progression [180, 181]. Notably, a PARP inhibitor may turn this chronic, low-level DNA stimulation into a more significant Th1 immune response [38, 182].

DDR inhibition may create a more sensitive TME by inhibiting Tregs. ATM activation is vitally important for tumor cell-derived small extracellular vesicles (sEV) to effectively induce Tregs (Fig. 2) [37]. ATM inhibitor KU-60019 treatment can reverse the phosphorylation of ATM and reduce the Treg ratio during sEVs stimulation [37]. However, olaparib administered in TNBC increases the level of Tregs, thus causing the CD4+/CD8+ ratio to increase [183]. However, cases have been reported where no significant change in Treg expression was observed when administering olaparib and a PD-1 antibody [170].

#### Innate immune cell

In addition to the aforementioned IFN pathway increasing NK cell and DC infiltration, several other mechanisms can also affect intrinsic cell infiltration. These processes involve changes on the surface of and within the immune cells.

In addition to NK cells that kill cancer cells [184, 185], innate immunity is inextricably involved in the antitumor effects associated with adaptive immunity. The upregulation of innate immunity promotes neoantigen presentation and enhances immune stimulation [185, 186]. From another perspective, intrinsic immune cells contribute to the adjuvanticity (as related to the antigenicity) of ICD and help form an activated immune microenvironment. Therefore, it is essential to consider the role of innate immune cell presentation in ICI responses.

##### NK cell

DDR inhibition affects NK and CD8 + T cell functions by regulating NKG2D/NKG2DL. The NKG2D receptor, which exists on NK cells and CD8+ CTL, combined with NKG2DL, can help drive innate immunity and support adaptive immunity (Fig. 2) [17]. NKG2D-CAR-T cell therapy targeting NKG2D ligands is at an early clinical stage [187]. The DNA damage observed leads to the up-regulation of the ligands of the NKG2D and DNAM-1 receptors. This damage also promotes the increase of IFN-γ secretion and stimulates both NK cells and CD8 + CTL [17, 44]. ATM inhibition prevents the expression of stress molecules on tumor cells [188]. Moreover, DDRi can delay NK cell failure and benefit NK cell proliferation, survival, and function [188]. The expression of NKG2DL in tumor cells may be the result of cGAS/STING pathway-induced IFN-1 expression (Fig. 2) [115].

Aging-related secretory phenotypes function to recruit innate immune cells, including NK cells and macrophages, as well as CD8 + T cells [127]. Instability of aneuploid accumulation after chromosome separation into the micronucleus can directly up-regulate senescence-associated secretory phenotypes (SASP) [189–191]. Since a large subset of SASP cytokines are dependent on DDR signaling for their production, SASP is considered to be the extracellular extension of DDR which affects the microenvironment via the paracrine signal [192–194]. Notably, IL-6 plays an important role in the promotion of cancer cell invasion by SASP [193]. Moreover, chronic signaling of cGAS/STING seems to promote the aging phenotype [192, 195–198].

##### Dendritic cell

DDR dysfunction-activated STING/IFN regulator 3 (IRF3) can lead to elevated DC levels [17, 44, 84, 199]. Type I interferon promotes the cross-presentation capability of DCs [84, 133]. In addition, compared with patients exhibiting < 2 DDR gene mutations, patients diagnosed with gastrointestinal cancer and ≥ 2 DDR gene mutations have been shown to express increased levels of effective immune cells, including activated dendritic cells, and decreased levels of immunosuppressive cells observed in tumor infiltration [47].

In a study of BRCA1-deficient ovarian cancer, dendritic cells expressed upregulated levels of CD80, CD86, and MHC II expression after olaparib treatment in comparison to the study’s vehicle control and the PD-1 antibody [170]. Moreover, an increase in CD103 + DC in the tumor tissue was observed, which reportedly stimulated effector T cell trafficking and T cell immune initiation [200].

HMGB1 is regarded as a relevant factor in ICD, and its effective role appears to be dependent on Toll-like receptor 4 (TLR-4) involvement (Fig. 2) [165]. The HMGB1 protein secreted by damaged apoptotic tumor cells can promote transcription of type I interferons (e.g., INF-β), as well as instigate DC maturation (Fig. 2) [201, 202]. DCs express IL-12, which allows Th1 cells to differentiate into Th1 effector cells and ultimately facilitates T-cell immunity [203].

##### Macrophage

In melanoma, ATR mutation is related to inhibitory TME features such as increased inhibitory macrophages. In addition, ATR inhibitors are beneficial to the response and effect of ICIs. Compared to the ATR wild-type subjects, the number of macrophages and B cells increased noticeably, and the number of CD3+ T cells decreased in the immune infiltration of ATR mutant melanomas [204]. Macrophages, which have been previously detected in genetically heterogeneous tumors, can promote melanoma invasion and metastasis [204]. In a Phase I trial of ceralasertib combined with paclitaxel, 11 out of 33 patients with melanoma experienced a reversal of the drug resistance to PD-1 therapy [205].

##### Myeloid-derived suppressor cell

The DDRi olaparib can successfully inhibit the recruitment of myeloid-derived suppressor cells (MDSCs) through the SDF1α/CXCR4 axis to improve the anti-tumor effect of CAR-T in mouse breast cancer [130]. Olaparib reduces the expression of SDF1α released by cancer-associated fibroblasts, thereby reducing CXCR4-regulated MDSC migration and effectively promoting CAR-T cell infiltration [130]. In addition, olaparib treatment in BRCA1-deficient ovarian cancer results in a decrease in granulocyte MDSCs [170].

##### Innate immune cell

One TCGA cohort analysis of cervical cancer proved that somatic DDR alterations were positively correlated with the characteristics and scores of hypoxia. And the high hypoxia scores were positively correlated with the abundance of resting mast cells, while the abundance of activated mast cells were low [142]. In HCC, the subtype of inhibitory DDR showed that mast cells and neutrophils related to ICB drug resistance were significantly enriched [71].

### Alteration of the DDR pathway affects immune regulatory molecules

The correlation between DDR changes, immune gene expression, and immune cycle steps has been verified in previous studies [56, 143, 171]. The level of DDR mutation and activation is related to enrichment of various immune features [34, 56]. These includes chemokines, lysis markers, MHC class II molecules, immune stimulators, and immune inhibitors [56]. High scores in DDR pathway activation were associated with both higher immune activation cell patterns and immune-related gene expression levels [34]. Interestingly, in muscle-invasive urothelial cancer, Thiago et al. analyzed the immune profiles of tumors harboring either biallelic, monoallelic, or wild-type mutations in the DDR genes. The expression of DDR genes and immunoregulatory genes were negatively correlated [171].

#### Pathway with cGAS/STING as the core

The cGAS/STING/IFNs pathway regulates innate and adaptive immunity. In tumors, defectiveness or inhibition of the DDR can lead to the accumulation of cytoplasmic DNA (Fig. 2) [44, 206, 207]. The sensing of cGAS to tumor-derived double-stranded DNA activates STING by generating cyclic GMP-AMP (cGAMP) (Fig. 2) [44, 208]. STING then induces a type I interferon reaction and consequently regulates the level of other immune and inflammatory factors (Fig. 2) [38, 44, 75, 208, 209]. PARPi, WEE1 inhibitor, ATM deficiency, and BRCA2 deficiency observed in tumors can promote both the recruitment of T cells, as well as the secretion of IFN-γ and TNFα in the TME through the cGAS/STING pathway (Fig. 2) [44, 114, 115, 124, 210–213]. This interferon-dependent pathway can activate both innate immunity and adaptive immunity, thus benefiting the remodeling of inhibitory TME [44, 212, 214]. In addition, the expression of PD-L1 is up-regulated at the transcription level [209], which is regarded as beneficial to ICIs.

There are several factors that can eliminate cytoplasmic nucleic acids in cancer cells. DDR factors, like RPA/RAD51, SAMHD1, and TREX1, can prevent excessive accumulation of cytoplasmic DNA [44, 76, 208, 215], while MRE11 can promote or inhibit the production of cytoplasmic DNA in different situations [44].

In this regard, STING is considered to be the focal point of the pathway. For example, PARPi promotes CAR-T infiltration in the TME via the cGAS-STING pathway. Specifically, PARPi promotes the upregulation of both IFN-β expression and chemokines via the STING-TBK1-IRF3 pathway, leading to CD8+ CAR-T cell recruitment and the secretion of large amounts of granzyme B [129]. This process ultimately allows low-dose CAR-T cell treatment to induce effective tumor regression in mice with kidney cancer [129]. DDR proteins ATM (Fig. 2), PARP1, DNA sensor (or DNA binding protein) IFI16, and Tp53 can also activate the STING pathway signal [76, 208, 216]. Moreover, MUS81 may help activate STING [44, 213].

Recently, researchers have found that the retinoic acid-inducible gene I (RIG-I)/mitochondrial antiviral signaling protein (MAVS) pathway has notable similarities to the cGAS/STING pathway (Fig. 2) [217, 218]. The interaction between the two pathways has been described in research [217].

#### ATM/ATR/Chk1 pathway

The ATM/ATR/Chk1 pathway regulates the cell cycle and can be activated by DNA damage signals [219–226], such as HR and NHEJ pathways, which are involved in DSB activation (Fig. 2) [209, 221].

ATM defective tumors can result in an increase in interferon signal transduction, innate immunity, and PD-L1 expression, all of which promote sensitivity to the PD-(L)1 blockade [227]. Specifically, ATM may inhibit SRC tyrosine kinase activity, while related ATM defects up-regulate SRC-promoted TBK1-PRR complex formation and type I interferon production independent of the cGAS/STING pathway [227, 228]. However, certain studies have shown that DSB activates ATM/ATR/Chk1, which can induce the activation of the classic STAT-IRF1 pathway. The inhibition of ATM or Chk1 can reduce IRF1 expression (Fig. 2) [221]. Furthermore, ATM can promote cancer cell migration by regulating the expression of IL-8 independent of its role in the repair of DNA double-strand breaks [229]. Co-inhibition of WEE1 combined with ATM reduced the expression of MMP-9, IL-8, CXCL1, CCL2, and CCL5, thus achieving an anti-migration effect in pancreatic cancer (PC) [230].

ATR mutation can regulate the tumor immune microenvironment in melanoma models and promote tumor growth [112, 204]. In previous studies, in addition to promoting T-cell homing to the epithelium, ATR mutant pigmented nevi also demonstrated a specific CD4 down-regulation effect [204, 231, 232]. Moreover, it is directly related to the increased expression of PD-L1 and CD206 [204], suggesting a relationship to the immune environment affiliated with T cell inhibition [112].

On the other hand, the effect of CHK1 inhibitors on interferon response is divergent. CHK1i can lead to an increase in cytoplasmic DNA, as well as the subsequent activation of the STING-TBK1-IRF3 pathway [9, 51]. As a result, both PD-L1 expression and type I interferon expression are upregulated. This results in the release of chemokines CXCL10 and CCL5 and facilitates the recruitment of CD8+ T cells [9]. CHK inhibitor prexasertib and anti-PD-L1 antibodies combination treatment can induce significant tumor regression in SCLC models [121]. However, Wayne et al. showed that CHK1i increased the phosphorylation of TBK1, but not the activation of IRF or type I interferon responses observed in solid cancer cell lines [131].

#### Abnormal DDR pathway and immune checkpoint molecules

The mutation of the DDR gene in cancer cells is usually associated with the increased expression of PD-1/PD-L1 genes within the TME [56, 64, 233, 234], such as the DSB repair gene [221], MMR gene [146, 235, 236] and RB1 [171, 237]. The expression of BER/SSBR has been shown to be negatively correlated with the level of PD-L1 observed in cancer cells [150, 235]. In addition, the clinical significance of PD-L1 in advanced GC depends specifically on the mutation of ARID1A and the level of ATM expression [238].

The following example illustrates the mechanism of the influence of DDR on PD-(L)1: the cGAS/STING pathway, activated by PARPi, leads to compensatory up-regulation of the PD-1/PD-L1 pathway [9, 118, 122]. This mechanism may be related to the secretion of IFN-γ, IFN-α, and IFN-β in the TME [211]. IFN-α and IFN-β can induce PD-L1 expression in endothelial cells, monocytes, and DC [209]. Glycogen synthase kinase 3β (GSK3β) subsequently interacts with PD-L1 and regulates its expression by inducing proteasome degradation of PD-L1 (Fig. 2) [38, 239]. PARPi deactivates GSK3β, thus enhancing the up-regulation of PD-L1 (Fig. 2) [33, 38, 122].

The ATM/ATR/CHK pathway is activated by DSB, such as when the BER/SSBR pathway is defective or during PARPi treatment, which in turn can up-regulate PD-L1 expression by phosphorylating STAT1/3 and therefore effectively inducing IRF1. It is also capable of up-regulating PD-L1 in circumstances that are independent of the IFN pathway (Fig. 2) [122, 136, 141, 150, 209, 221, 240–244]. Some studies have shown that the ATM/ATR/Chk1 pathway can up-regulate PD-L1 by activating the STAT1/3-IRF1 pathway in cancer cells (Fig. 2) [221, 245]. Up-regulation of PD-L1 expression induced by activating IR is carried out through transcription and post-translation mechanisms, while the ATM/BCLAF1/PD-L1 axis regulates the stability of PD-L1 in response to IR [246].

The inhibition of specific components in the ATM/ATR/Chk1 pathway can cause various effects on the stability of PD-L1. For instance, increased expression of PD-L1 can be observed in ATM-defective tumors [227]. However, another study investigating pancreatic cancer cells revealed that the inhibition of WEE1/ATM could effectively down-regulate the expression of PD-L1 by blocking GSK-3β and reducing the expression of CMTM6 [230]. ATM can also up-regulate PD-L1 through the JAK-STAT3 pathway, and inhibition of ATM can reduce PD-L1 expression [245]. Furthermore, while ATR inhibitor (ATRi) up-regulated PD-L1 mRNA in cells, it also led to the activation of the CDK1-SPOP axis and subsequent degradation of PD-L1. ATRi/ATR siRNA treatment led to the downregulation of PD-L1 protein expression, while, conversely, olaparib increased the expression of the PD-L1 protein [10, 122]. According to previous studies, post-translational regulation may in fact serve to play a major role in the regulation of PD-L1 by ATR [219]. Similarly, CHK1i activates the STING/TBK1/IRF3 innate immune pathway and increases the expression of PD-L1, as well as other immune infiltration changes [114, 211].

The alteration of the DDR pathway also affects other immune checkpoints, as has been seen when the expression of CTLA-4 and other immune checkpoints in the dMMR tumor increased as a result [146, 235]. IFN1 signaling can increase the molecular level of checkpoints, such as indoleamine 2,3-dioxygenase 1 (IDO1) and lymphocyte activation gene 3 protein (LAG3), in various immune cells [171]. The high expression of BRCA1 in tumor cells is significantly related to the positive expression of the new target Siglec-15 and a favorable prognosis [247, 248].

## Prospects

At present, expanding the use of immune checkpoint inhibitors is a matter of great urgency. DDR displays great potential in this regard [249], as the alteration of the DDR pathway in tumors as a predictive marker of ICB presents many advantages [20, 249, 250]. More importantly, the benefits of DDR inhibitors have been extended to other immunotherapies beyond ICB [130]. Although the role of the DDR pathway dysfunction in tumor progression and immune regulation is somewhat uncertain [58, 112, 251] and not always useful [58, 83, 110, 115, 181].

As the anti-tumor benefits generated by DDR functional deficiency are deserving of greater research attention, we have put forward here several research directions and suggestions regarding the application of the DDR pathway in ICB. (1) DDR pathways require improved detection methods. This may entail devising novel strategies that dynamically detect DNA repair function and essentially avoid the associated errors caused by the time heterogeneity of somatic mutations [58]. (2) It is necessary to determine reliable predictive biomarkers, pay more attention to the prediction results of the DDR pathway co-mutation, and determine the appropriate unified labeling threshold [87, 115]. (3) Emphasis should be placed on investigative efforts aimed at defining the predictive role of the overall activation level of the DDR pathway. The first large GC cohort to investigate DDR pathway activity proved the clinical transformation value of the DDR pathway spectrum for patients. Patients with low DDR pathway signature scores might not benefit from anti-PD1 therapy [80]. (4) Increased research is advised regarding combination therapy, including the combination of two DDR inhibitors and immunotherapy. Moreover, the synergistic association of DDR inhibitors with certain defects may achieve more effective and accurate immunomodulation [213]. For example, ATM inhibitors are used in ATM defective tumors with positive effect [252]. Meanwhile, drug selection, dosage, and combination time are all carefully considered during personalized treatment, in addition to toxicity, immune-related adverse events, and drug resistance [112, 115]. Tumor type should also be considered in combination therapy. For example, the characteristics of the TNBC make it advantageous for combined therapy [93], while the determinants of ICB activity in SCLC remain unclear [121]. Ultimately, more randomized clinical trials are required to determine whether these combined treatments are superior to ICI alone, which must also be verified in larger clinical cohorts [58, 115]. (5) While there are limited reports exploring specific genes, such as the NHEJ and NER pathway, the relationship between other DDR pathways and immunotherapy remains to be investigated [58]. (6) To the best of our knowledge, the mechanism of DDR’s influence on the immune microenvironment is primarily focused on tumor cells rather than immune cells, though this particular aspect requires more in-depth study. (7) Further exploration of the relationship between emerging immune checkpoints and DDR pathway alterations is required. New ICIs are one of the primary hopes of patients to improve their OS when they fail to exhibit any response to anti-PD-L1 treatment. However, this topic has considerable space for development [248]. For example, the positive expression of Siglec-15 is significantly correlated with the high expression of BRCA1, but its mechanism has not yet been clarified.

## Conclusions

DDR factors are competitive predictive biomarkers of specific ICI responses that have become increasingly relevant in current research. In addition to the more established dMMR, the primary focus of scholarship on this topic thus far has been investigating the predictive effects of combined DDR mutations. The combination of PARPi and anti-PD-L1 mAbs has yielded positive results, though more DDRi combination treatments for ICIs are required in current preclinical trials. Defection or inhibition of the DDR pathway can increase the efficacy of immunotherapy by increasing immunogenicity due to aggravated DNA damage, thus inhibiting the DNA damage sensing pathway and causing the accumulation of cytoplasmic DNA to activate the IFN pathway. However, the effect of DDRi is not consistent. Therefore, the choice of combination therapy should not necessarily be recommended for all cancer types.

## Data Availability

Data sharing is not applicable to this article as no datasets were generated or analysed during the current study.

## Abbreviations

AMPK: AMP-activated protein kinase
ARID1A: AT-rich interaction domain 1A
ATM: Ataxia-telangiectasia mutated
ATR: Ataxia telangiectasia and Rad3-related protein
ATRi: ATR inhibitor
BAP1: BRCA1 associated protein 1
BER: Base excision repair
BRCA1/2: Breast cancer susceptibility gene 1/2
CAR-T: Chimeric antigen receptor T cell
CCL2: C-C motif chemokine ligand 2
CCL5: C-C motif chemokine ligand 5
cGAMP: Cyclic GMP-AMP
cGAS: Cyclic GMP-AMP synthase
CHK: Checkpoint kinase
CHK1i: CHK1 inhibitor
CMTM6: CKLF-like MARVEL transmembrane domain containing 6
Co-mut-: Co-mutation negative
Co-mut+: Co-mutation positive
CTL: Cytotoxic T lymphocyte
CTLA-4: Cytotoxic T-lymphocyte-associated protein 4
CXCL1: C-X-C motif chemokine ligand 1
CXCL10: C-X-C motif chemokine ligand 10
CXCL11: C-X-C motif chemokine ligand 11
CXCL9: C-X-C motif chemokine ligand 9
CXCR4: C-X-C motif chemokine receptor 4
DAMPs: Damage-associated molecular patterns
DCB: Durable clinical benefit
DDR: DNA damage repair
DDR1: The DDR-related gene low expression group
DDR2: The high expression of DDR-related genes
DDRi: DDR inhibitors
dMMR: Mismatch repair-deficient
DNAM-1: DNAX accessory molecule-1, CD226
DSB: DNA double strand break
DSSH: DDR ssGSEA enrichment score-high
FOXP3: Forkhead box P3
GC: Gastric cancer
GSK3β: Glycogen synthase kinase 3β
HCC: Hepatocellular carcinoma
HMGB1: High mobility group box 1
HR: Homologous recombination
HRD: Homologous recombination deficiency
HRR: Homologous recombination repair
ICB: Immune checkpoint blockade
ICD: Immunogenic cell death
ICIs: Immune checkpoint inhibitors
IDO1: Indoleamine 2,3-dioxygenase 1
IFI16: Interferon-gamma-inducible protein 16
IFN: Interferon
IFN1: Interferon type I
IL-1: Interleukin 1
IL-6: Interleukin 6
IL-8: Interleukin-8,C-X-C motif chemokine ligand 8
IRF1: Interferon regulatory factor 1
IRF3: IFN regulator 3
JAK2: Janus kinase 2
LAG3: Lymphocyte activation gene 3 protein
MAMPs: Microorganism-associated molecular patterns
MAVS: Mitochondrial antiviral signaling protein
mCRPC: Metastatic castration-resistant prostate cancer
MDSCs: Myeloid-derived suppressor cells
MHC: Major histocompatibility complex
MMP-9: Matrix metalloproteinase-9
MMR: Mismatch repair
MRE11: Meiotic recombination 11 homolog, double strand break repair nuclease
MSI: Microsatellite instability
MSI-H: Microsatellite instability-high
MSS: Microsatellite stable
MUS81: Methyl methanesulphonate and ultraviolet-sensitive 81, structure-specific endonuclease subunit
MyD88: Myeloid differentiation factor 88
NAL: Neoantigen load
NER: Nucleotide excision repair
NHEJ: Non-homologous end joining
NK cells: Natural killer cells
NKG2D: Natural-killer group 2, member D
NKG2DL: Ligands for the receptors NKG2D
NSCLC: Non-small cell lung cancer
ORR: Objective response rate
OS: Overall survival
PARP: Poly (ADP-ribose) polymerase
PARPi: PARP inhibitors
PC: Pancreatic cancer
PD-1: Programmed cell death protein 1
PD-L1: Programmed cell death 1 ligand 1
PFS: Progression-free survival
POLD1: DNA Polymerase Delta 1, catalytic subunit
POLE: DNA Polymerase Epsilon, catalytic subunit
PRR: Pattern recognition receptor
RAD51: RAD51 recombinase
RB1: RB Transcriptional Corepressor 1
RIG-I: Retinoic acid-inducible gene I
RPA: Replication protein A
SAMHD1: Sterile alpha motif and HD domain containing protein 1
SASP: Senescence-associated secretory phenotype
SCLC: Small cell lung cancer
SDF1α: Stromal cell-derived factor 1 alpha, C-X-C motif chemokine ligand 12 alpha
sEV: Small extracellular vesicles
Siglec-15: Sialic acid-binding immunoglobulin-like lectin 15
SIRT1: Sirtuin 1
SIRT2: Sirtuin 2
SIRT6: Sirtuin 6
SRC: SRC tyrosine kinase
SSBR: Single strand break repair
STAT1: Signal transducer and activator of transcription 1
STAT3: Signal transducer and activator of transcription 3
STING: Stimulator of interferon genes
TBK1: TANK binding kinase 1
TCGA: The Cancer Genome Atlas
TILs: Tumor infiltrating lymphocytes
TLR-4: Toll-like receptor 4
TMB: Tumor mutational burden
TMB-H: Tumor mutational burden-high
TME: Tumor microenvironment
TNBC: Triple negative breast cancer
TNF-α: Tumor necrosis factor α
TP53: Tumor protein P53
Treg: Regulatory T cell
TREX1: Three-prime repair exonuclease 1
INF-β/TRIF: Toll/IL-1 receptor (TIR) domain-containing adaptor-inducing interferon-β
UC: Urothelial carcinoma
WEE1: WEE1 G2 checkpoint kinase
XRCC1: X-ray cross complementing group 1
